# Effect of the Extraction Process on the Biological Activity of Lyophilized Apricot Extracts Recovered from Apricot Pomace

**DOI:** 10.3390/antiox7010011

**Published:** 2018-01-14

**Authors:** Dina Cheaib, Nada El Darra, Hiba N. Rajha, Iman El-Ghazzawi, Richard G. Maroun, Nicolas Louka

**Affiliations:** 1 Faculty of Sciences, Beirut Arab University, P.O. Box 115020, Riad El Solh, Beirut 1107 2809, Lebanon; dac273@student.bau.edu.lb; 2 Faculty of Health Sciences, Beirut Arab University, Tarik El Jedidah—Beirut, P.O. Box 115020, Riad El Solh, Beirut 1107 2809, Lebanon; i.ghazzawi@bau.edu.lb; 3 Unité de Recherche Technologies et Valorisation Agro-alimentaire, Centre d’Analyses et de Recherche, Faculté des Sciences, Université Saint-Joseph de Beyrouth, B.P. 11-514 Riad El Solh, Beirut 1107 2050, Lebanon; hiba.rajha@usj.edu.lb (H.N.R.); richard.maroun@usj.edu.lb (R.G.M.); nicolas.louka@usj.edu.lb (N.L.)

**Keywords:** lyophilization, apricot pomace, heat-assisted extraction, infrared, polyphenols

## Abstract

The preservation of polyphenols in fruits by lyophilization has gained great interest in the recent decades. The present study aims to assess the impact of the pre-treatment extraction methods heat-assisted extraction (HAE) and infrared (IR) on lyophilized apricot pomace extracts. Then to test the conservation of polyphenols quantities as well as their bioactivities (antiradical and antibacterial) in lyophilized extract. An aqueous extract was obtained through either heat-assisted extraction or infrared pre-treatments then lyophilized to obtain a dried form. Results showed that the content of polyphenols, the antiradical and antibacterial activities in lyophilized extracts exhibited a slighter decrease in infrared sample compared to the heat-assisted extraction ones. The High-performance liquid chromatography (HPLC) analysis showed that lyophilized extracts IR and HAE preserved the same phenolic molecules (rutin, catechin and epicatechin) detected in liquid extracts (IR and HAE) with a smaller yield. Lyophilization can be used as a widely process in the food industry to conserve many bioactive molecules.

## 1. Introduction

In the last decade, phenolic compounds have gained a considerable interest due to their different biological properties [1]. They are closely linked with the nutritional and sensory quality of fruits and vegetables [2,3]. The phenolic composition and the biological properties of fruits can be affected by different factors such as variety, climate, harvest time, storage as well as post-harvest processes like drying, cutting, storage, packaging, fermentation, and cooking processing [4,5]. In order to preserve the nutritional components of fruits (such as vitamins and carotenoids), it is generally recommended to dry product by lyophilization or (freeze-drying). The latter is known to be an effective method used to preserve a wide variety of materials (such as proteins, microbes, tissues and plasma), produce commercial products (pharmaceutical, natural additives, and preservatives for food industries) and to extend the shelf-life of foods by preventing the microbial growth, inhibiting enzymatic degradation, and retarding lipid oxidation [6,7,8,9]. On the other hand, during the lyophilization process, there might be a chance of decrease in the antioxidant property caused by the degradation of certain bioactive compounds (such as phenolic compounds, ascorbic acid) [10,11]. In order to protect polyphenols from freeze-drying, a maltodextrin precoating process may be applied before the drying process [12]. Many studies compared the nutritional composition of fresh and freeze-dried fruits and vegetables. A loss of certain vitamins (vitamins A and C) was noted, as well as a decrease in the nutritional value in some vegetables due to the freeze-drying process [9,10,13]. Moreover, a decrease in total phenolic content in freeze-dried mango fruits compared to fresh ones (from 14 to 17 mg gallic acid equivalent (GAE)/100 g dry sample) was showed by Rahman et al. (2015) [14]. However, the comparison between liquid and lyophilized extract compositions obtained by different extraction techniques have not been well explored. The infrared-assisted extraction (IRAE) technique (with wavelength range between 1.72 and 16.66 μm) is conducted with an infrared lamp. IRAE is used as a heat source due to its highest efficiency when its wavelength matches the absorption properties of the material it is warming [15,16]. Compared to other conventional techniques (heat assisted extraction and microwave assisted extraction), infrared is easy to use and could enhance the extraction yield of many bioactive molecules from medicinal plants [17].

Due to the diverse application fields of phenolic compounds, their extraction and isolation from fruits byproducts have been widely studied. However, the impact of extraction methods (as a pre-treatment) on the lyophilization process has not been yet documented.

The present work aims first to study the impact of the extraction methods; heat-assisted extraction (HAE) and infrared (IR) on lyophilized apricot pomace extracts and second, to test the conservation of polyphenols quantities as well as their bioactivities (antiradical and antibacterial) in lyophilized extract (powder-dried form).

## 2. Materials and Methods

### 2.1. Raw Materials

Apricot pomace was obtained from Conserves Modernes Chtaura (Chtaura, Lebanon); this industry is specialized in the production of fruit purees and jams. The pomace is made of pressed skins and pulp residues (of fresh apricots).

### 2.2. Extraction Experiments

The extraction conditions were conducted at different durations in order to reach the same energy consumption of 2290.90 kJ/kg for the infrared and the heat-assisted extraction methods.

#### 2.2.1. Infrared-Assisted Extraction

The infrared-assisted extraction apparatus was designed at Faculté des Sciences, Université Saint Joseph de Beyrouth (Beirut, Lebanon). It consists of a ceramic infrared transmitter with a power between 63 and 170 W to conduct the heating process connected to a proportional–integral–derivative (PID) control and temperature adjustment system. The temperature and the voltage can be regulated manually or automatically. This apparatus utilize infrared energy from the ceramic to heat the solvent sample mixture placed in a round bottom flask and set at a variable distance away from the ceramic transmitter. An amount of 10 g of apricot pomace was mixed with 100 mL of preheated water (75 °C) in the round-bottom flask and placed on the top of the ceramic transmitter. The temperature was fixed at 75 °C for 60 min (according to a preliminary study). The infrared (IR) energy $W_{IR}\;\left(\mathrm{kJ}/\mathrm{kg}\right)$ was calculated:

(1) $$W_{IR}=\frac{P_{IR}\times t_{IR}}{m}$$

where *P_IR_* is the generator power (70 W), $t_{IR}$ is the total extraction duration (s), and *m* is the product mass (g). Note that *m* = 110 g (100 g water + 10 g dry matter) and *W_IR_* is the energy consumed (2290.90 kJ/kg).

#### 2.2.2. Heat-Assisted Extraction

The heat-assisted extraction was conducted using a water bath with a power of 660 W. The glass flask containing 10 g of apricot pomace mixed in 100 mL of preheated water at 75 °C, and then covered by aluminum foil in order to avoid polyphenol degradation. The temperature of the water bath was fixed at 75 °C.

The time required for the heat-assisted extraction $t_{HAE}\;\left(s\right)$ to reach the same energy of infrared technique was calculated as follows:

(2) $$t_{HAE}=\frac{W_{HAE}\times m}{P_{HAE}}$$

where *W_HAE_* is the energy consumed (2290.90 kJ/kg), *P_HAE_* is the generator power (660 W), *m* is the product mass (g) and $t_{HAE}$ is the total extraction duration (s) (which is 6 min).

The extracts were then filtered using glass wool before analysis.

### 2.3. Lyophilization

Liquid samples were placed in petri dishes (7 mm), then frozen at −20 °C and finally lyophilized at −55 °C under vacuum (Ilshin Lab. Co., Ltd., Yangju-si, Korea) for 48 h.

### 2.4. Analysis

#### 2.4.1. Dry Matter Content

The analysis of the dry matter content of the raw material was carried out by weighing an specific amount of sample and drying it for 24 h using a ventilated oven at 105 °C [18].

#### 2.4.2. Quantification of Total Polyphenol Content by Folin-Ciocalteu Method

In accordance with the Folin Ciocalteu (FC) method, 0.2 mL of the extract, 0.1 mL of commercial FC reagent and 0.8 mL of sodium carbonate solution (75 mg/L) were mixed and kept for 10 min at 60 °C [19]. The absorbance of the extracts was then read at 750 nm by a spectrophotometer UV-VIS (Gold S54T UV-VIS, Shanghai, China). The absorbance values were compared to a standard curve of prepared gallic acid solutions and expressed as milligram of gallic acid equivalents (GAE) per gram (g) of dry matter (mg GAE/g DM).

#### 2.4.3. Determination of the Antiradical Activity

The free radical scavenging activity was determined by the capacity of the phenolic molecules in the samples to reduce DPPH (2,2-diphenyl-picrylhydrazyl), a stable free radical. In brief, 4 mL of 1 mM DPPH was mixed with 0.2 mL of the extract. The absorbance at 517 nm of the extracts was measured after 30 min at room temperature using methanol as a blank. The decrease in the absorbance allowed the calculation of the inhibition percentage. Two models were represented; the percentage of inhibition at the initial concentration of the extracts and the inhibition percentage at a fixed concentration of polyphenols (2.7 mg GAE/g DM) in order to determine the influence of their diversity [20].

#### 2.4.4. HPLC-DAD Analysis

Polyphenol analyses of the liquid extracts and lyophilized dried from prepared from apricot pomace were performed by High-Performance Liquid Chromatography (HPLC). Prior to analytical chromatography.

The samples were purified by filtration through 0.2 µm syringe filters to remove interferences of sugars and organic acids from the crude samples. An equipment consisting of liquid-chromatography-Agilent 1100 Series system (Teknokroma Professional Friendly Lichrospher 100 RP18 5 µM, 25 × 0.46, Serial number NF-21378, Barcelona, Spain), equipped with an autosampler, a Zorbax column oven (Barcelona, Spain), coupled to a diode array detector. The separation of polyphenols was performed through a C18 Column (25 × 0.46 mm). The standards used for identification and quantification purchased from Sigma (Aldrich) were: trans-cinnamic acid, caffeic acid, epicatechin, hydroxybenzoic acid, chlorogenic acid, catechin, rutin, gallic acid, kaempferol, catechin gallate and myrecetin (purity ≥ 98%). The mobile phase consisted of acidified nanopure water at pH 2.3 with HCl (A) and acetonitrile (B) HPLC grade. The elution was done under isocratic conditions from 0 to 5 min with (85%) A and (15%) B. Gradient profile was from 5 to 30 min, beginning with (85%) A and (15%) B and ending with (0%) A and (100%) B. It was followed by isocratic conditions from 30 to 35 min with (0%) A and (100%) B. The injection volume was 10 µL and the flow rate was 1 mL/min. Based on the retention time and the spectra of the original standard each peak was identified. Quantification was accomplished using the phenolic standards solutions [21].

#### 2.4.5. Determination of the Antibacterial Activity

##### Bacterial Strains, Culture Media and Growth Conditions

For the antibacterial activity; eight clinical bacterial strains were studied: two strains of *Staphylococcus aureus*, amongst which, one is *Methicillin-Resistant Staphylococcus Aureus* (MRSA 3); one strain of *Coagulase-negative Staphylococci* (1530), one strain of *Staphylococcus epidermidis* (2080), two strains of *Klebsiella*, amongst which, one is Metallo-beta-lactamase producer and two strains of *Escherichia coli* amongst which, one is extended spectrum beta lactamase (ESBL 2238).

##### Preparation of the Bacterial Inocula for Minimum Inhibitory Concentration (MIC)

All strains were subcultured using freshly-prepared blood agar. Part of five colonies of each strain were inoculated in 3 mL of cation adjusted Mueller Hinton broth. After reaching a turbidity of 0.5 McFarland, a dilution of 1/100 was made into tubes of adjusted Mueller Hinton broth.

##### Phenolic Extracts Preparation for MIC Assessment

Five serial dilutions of each extracts (infrared and solid-liquid) were conducted (starting from 1.6 to 26 µg/mL). Before the analysis, all stocks were sterilized using 0.4 µm disposable syringe filters. After the addition of equal volumes (100 µL) of each concentration of the four extracts to the bacterial strains (100 µL) in a 96 well (U shape) microtiter plates, the final concentrations of the different phenolic extracts were reduced to: 13.5 µg/mL up to 0.8 µg/mL. Two plates were utilized in this experiment. Finally, the plates of each of the phenolic extracts were read for the MIC that inhibits the bacterial growth after an overnight incubation at 37 °C.

#### 2.4.6. Statistical Analysis

Each experiment was conducted in duplicates and analysis repeated twice. Means and standard deviations of data were calculated. The error bars in all figures correspond to the standard errors. Variance analyses (ANOVA) and Least Significant Difference test (LSD) were conducted to evaluate the significant differences between the results. STATGRAPHICS^®^ Centurion XV (StatPoint Technologies, Inc., Warrenton, VA, USA) was used to perform statistical analysis.

## 3. Results and Discussion

### 3.1. Total Polyphenols Content

**Figure 1:**
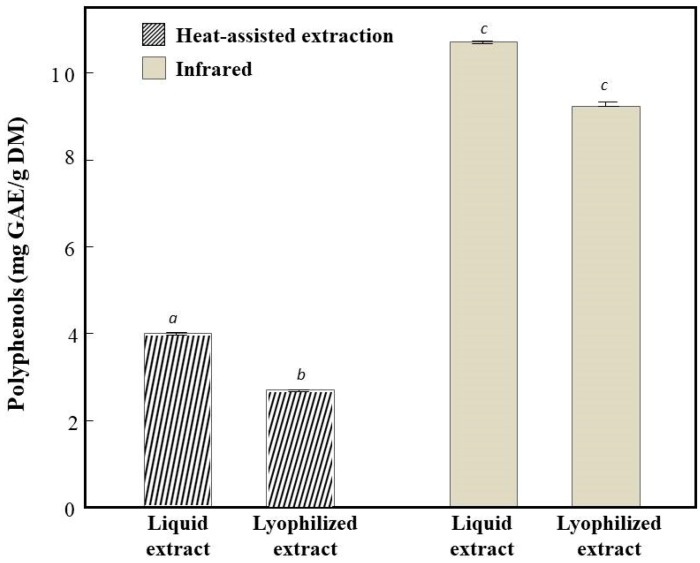
Polyphenol content of heat-assisted extraction and infrared apricot pomace extracts (liquid and lyophilized). Different superscript letters indicate significant statistical difference (*p* < 0.05).

Figure 1 represents the total phenolic content of apricot pomace extracts liquid and lyophilized (HAE and IR). IR lyophilized gave higher phenolic content compared to HAE lyophilized. On the other hand HAE lyophilized showed a decrease of 32% (ranging from 4 to 2.7 mg GAE/g DM) of phenolic content compared to HAE liquid extract while IR lyophilized showed a slight decrease 16% (ranging from 10.8 to 9 mg GAE/g DM) of the total phenolic content compared to IR liquid extract. Many studies showed a reduction in polyphenolic yield from lyophilized fruits and vegetables compared to fresh ones. Georgé et al. (2010) found that the total polyphenol content of yellow tomatoes, decreased by 30% in lyophilized tomato compared to fresh ones [22]. Moreover the level of phenolic compounds in some tropical fruits (watermelon, papaya, mango and melon) decreased by 48.23%, 39.73%, 23.19% and 10.41% respectively after freeze drying process [10].

This decrease in the polyphenols content in lyophilized fruits and vegetables products could be due to the rupture of the cell wall caused by ice crystals formation and the subsequent release of hydrolytic and oxidative enzymes (which are not fully deactivated) which cause a degradation in phenolic compounds [22,23,24]. However, in our study a liquid extract was subjected to lyophilization and, thus, no cells were found in the lyophilized sample. This decrease in polyphenolic yield in lyophilized extract could be attributed to the changes in the structure of the extracted molecules (phenolic compounds) which was reported by Deladino et al. (2013) who showed a reduction in polyphenol content by 20% in lyophilized extract compared to liquid one from Yerba mate (*Ilex paraguariensis*) [25]. Although freeze-drying appears to be an effective method of preservation, its effect on polyphenolic molecules is variable depending on the method of extraction, type of solvent used and sample matrix [26]. Regarding the extraction technique, the slight decrease of phenolic content in IR lyophilized compared to the HAE one could be explained by the mode of action of the extraction method which affects the cellular structure differently. IR radiation directly heats the sample matrix which could extract more bioactive compounds, while in conventional techniques more time is required to heat the vessel before the energy of heating is transferred to the sample mixture [15]. Moreover, the efficiency of the IR recovery can be attributed to the matching between the infrared wavelength and the absorption characteristics of the solvent [17].

### 3.2. Biological Activities

#### 3.2.1. Antiradical Activity (AA)

**Figure 2:**
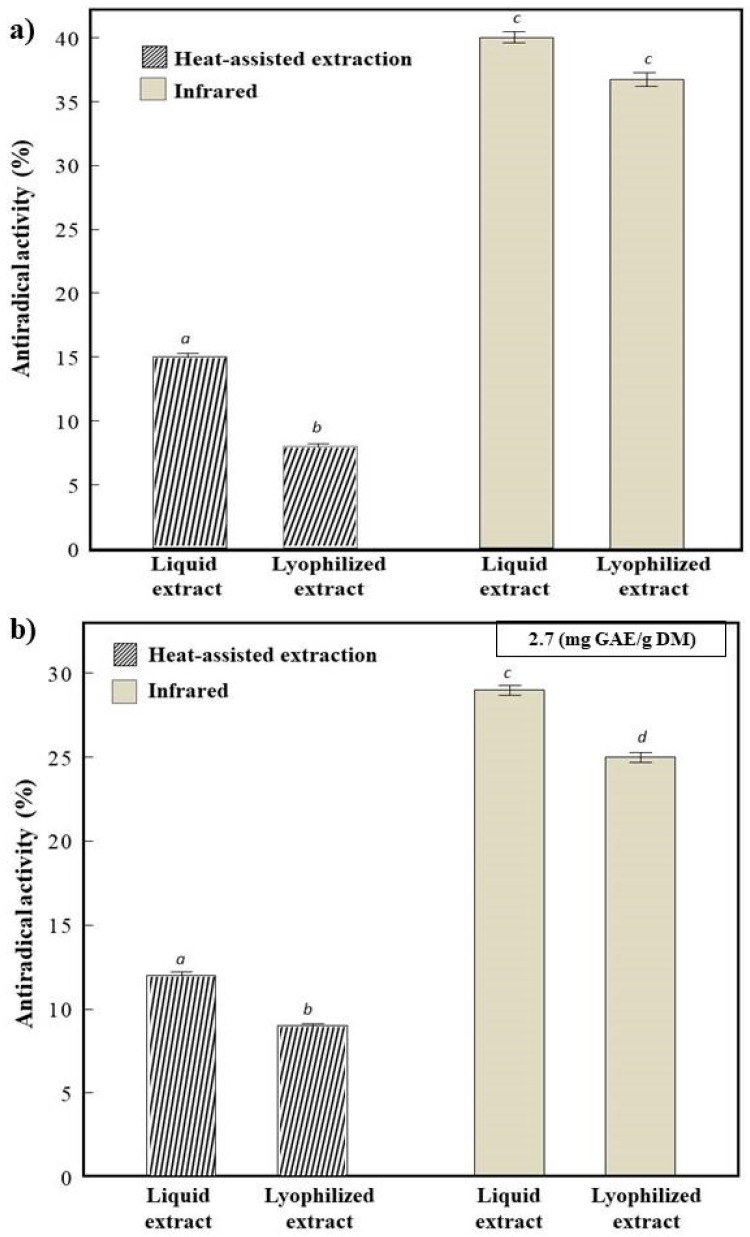
Antiradical activity of heat-assisted extraction and infrared apricot pomace extracts (liquid and lyophilized). (**a**) at their initial concentration and (**b**) at 2.7 mg GAE/g DM of polyphenols. Different superscript letters indicate significant statistical difference (*p* < 0.05).

Figure 2a represents the antiradical activity (AA) of apricot pomace liquid extracts and lyophilized (HAE and IR) at their initial concentration. HAE lyophilized showed a decrease of 46% of AA compared to HAE liquid extract, while IR lyophilized showed a 10% decrease compared to IR liquid extract. These findings could be correlated to the higher decrease in polyphenols content in HAE lyophilized compared to IR lyophilized (Figure 1). On the other hand, at the same polyphenol concentration (Figure 2b), IR lyophilized exhibited a better AA than HAE lyophilized sample. These results could be attributed to the different polyphenol composition, responsible for the enhancement of the radical scavenging activity in each method of extraction [27]. To confirm this hypothesis, HPLC analysis was conducted in our study to identify the phenolic compounds responsible for the antiradical activity. The antiradical activity results showed that lyophilized sample exhibited an AA, which was more conserved in the infrared sample than in the heat-assisted extraction extract.

#### 3.2.2. Antibacterial Activity of the Apricot Pomace Liquid and Lyophilized Extracts

The antibacterial activity of apricot pomace liquid and lyophilized extracts treated by HAE and IR technique were tested against different Gram-positive strains and Gram-negative bacterial strains (Table 1) using MIC for the different concentrations 13.5 µg/mL, 6.75 µg/mL, 3.3 µg/mL, 1.6 µg/mL, and 0.8 µg/mL. HAE and IR lyophilized samples showed antibacterial activity. IR liquid extract exhibited the lowest inhibitory concentration denoting the highest inhibitory bacterial activity against all Gram-positive strains and one Gram-negative strain. IR lyophilized showed antibacterial activity against the same bacterial strains tested for IR liquid but the minimum inhibitory concentration (MIC) was higher for the IR lyophilized. On the other hand, HAE (liquid and lyophilized extracts) showed the highest MIC exhibiting, the lowest inhibition against only two Gram-positive in comparison with IR liquid and lyophilized extracts respectively. The antibacterial results are in concordance with the radical scavenging activity, where the IR liquid extract gave the highest activity followed by IR lyophilized and HAE liquid extract. The HAE lyophilized gave the lowest antibacterial activity. Table 1 showed that the effect of polyphenols was only against one Gram-negative bacterial strain compared to Gram-positive ones. Those results can be explained by the fact that phenolic molecules have higher activity against gram-positive bacteria in comparison to gram-negative ones. The latter have an outer membrane in their cell wall which plays a role of a barrier and thus decreasing the uptake. Moreover, the reduction of the activity of polyphenols in some bacteria could be also due to other mechanism of resistance such as the mutation in porin protein or the efflux phenomena [28,29]. Our findings showed that lyophilized samples preserved their antibacterial activity with increased inhibition concentrations compared to (IR and HAE) liquid extracts.

### 3.3. Quantification of Polyphenol Extracts by High-Performance Liquid Chromatography

**Figure 3:**
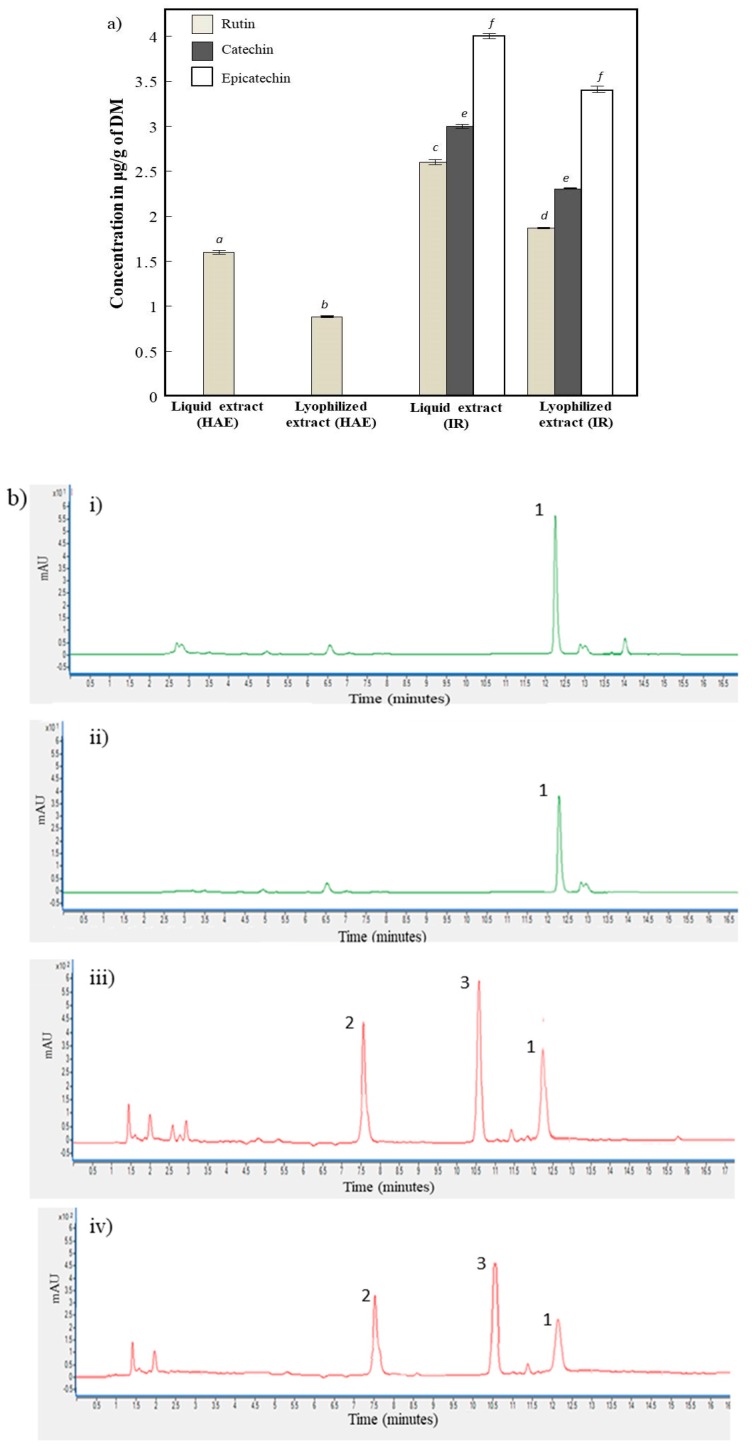
(**a**) Polyphenols composition of heat-assisted extraction and infrared apricot pomace extracts (liquid and lyophilized). Different superscript letters indicate significant statistical difference (*p* < 0.05). **(b)** Chromatographic profile of the several phenolic compounds 1: Rutin, 2: catechin and 3: epicatechin of: i: liquid HAE, ii: lyophilized HAE, iii: liquid IR and iv: lyophilized IR.

The composition of polyphenol in apricot pomace extracts and lyophilized (IR and HAE) was shown in Figure 3a. The chromatographic profile of the several phenolic compounds from different extracts was shown in Figure 3b. The main polyphenols were rutin, catechin and epicatechin which was consistent with the results of Veberic and Stampar (2005) on the polyphenol composition in apricot varieties [30]. Rutin was found in all samples. However, catechin and epicatechin were only detected in infrared liquid and lyophilized extracts suggesting the selectivity of IR towards those compounds. IR liquid and lyophilized extracts showed the highest extraction yield for all the detected polyphenol compounds compared to HAE extract and HAE lyophilized respectively.

Figure 3 showed that lyophilized samples (IR and HAE) preserved the same phenolic molecules detected in liquid extracts (IR and HAE) with a smaller yield. Rutin in HAE lyophilized decreased by 45% compared to IR lyophilized which showed a reduction of 34%. Our results are in accordance with those of Lorena Deladino et al. (2013) who found that the amounts of rutin in the lyophilized sample was lower (by 28%) than in the liquid extract from Yerba mate [25]. Concerning catechin and epicatechin, their concentrations decreased by 26% and 22% in IR lyophilized, respectively. Those results showed that lyophilization process depends not only on the technique of extraction (pretreatment) but also on the structure of the extracted molecules [22]. The Freeze-drying process can alter some phenolic compounds (rutin) more than others (catechin and epicatechin) as shown in Figure 3.

## 4. Conclusions

In conclusion, the content of polyphenols and the antiradical and antibacterial activities in the lyophilized samples showed a slighter decrease in infrared samples than in heat-assisted extraction ones. After lyophilization process, the same phenolic compounds were identified in lyophilized extracts, which could explain the preservation of their biological activities especially in infrared samples. The latter seems to better preserve the structure of the extracted molecules in comparison to heat assisted extraction method which showed more decrease in polyphenol yield. This difference in polyphenolic content could be due to mode of action of each technique; the heat-assisted extraction method seems to fragilize more the extracted molecules which become more vulnerable to lyophilization treatment. Since the freeze-drying process is a widely used technique to stabilize plant material over a long duration, it could be of interest to determine the factors or mechanisms that could affect the phenolic content and biological activities during this process.

## Figures and Tables

**Table 1:** Minimum inhibitory concentration (µg/mL) of different Gram-positive and Gram-negative bacteria from heat-assisted extraction (HAE) and infrared (IR) apricot pomace extracts (liquid and lyophilized).

Minimum Inhibitory Concentration (µg/mL)				
Bacterica/POMs	Heat-Assisted Extraction		Infrared	
	Liquid Extract	Lyophilized Extract	Liquid Extract	Lyophilized Extract
Methicillin Resistant Staphylococcus aureus (MRSA3) (gram+)	-	-	6.75	13.5
Staphylococcus aureus 1966 (gram+)	6.75	13.5	3.3	6.75
Coagulase-negative Staphylococci 1530 (gram+)	-	-	3.3	6.75
Staphylococcus epidermidis 2080 (gram+)	6.75	13.5	3.3	6.75
Klebsiella 118 metallo beta lactamase + (gram−)	-	-	-	-
Klebsiella (gram−)	-	-	-	-
*E. coli* ESBL 2238 (gram−)	-	-	-	-
*Escherichia coli* (1250) (gram−)	-	-	6.75	13.5

- indicates absence of antibacterial activity.
